# From Bench to Bedside in Tongue Muscle Cancer Invasion and Back again: Gross Anatomy, Microanatomy, Surgical Treatments and Basic Research

**DOI:** 10.3390/life10090197

**Published:** 2020-09-12

**Authors:** Luca Calabrese, Maria Eleonora Bizzoca, Roberto Grigolato, Fausto Antonio Maffini, Marta Tagliabue, Rosa Negro, Stefania Leuci, Michele Davide Mignogna, Lorenzo Lo Muzio

**Affiliations:** 1 Division of Otorhinolaryngology, San Maurizio Hospital, 39100 Bolzano, Italy; dott.lucacalabrese@gmail.com; 2 Department of Clinical and Experimental Medicine, University of Foggia, 71122 Foggia, Italy; marielebizzoca@gmail.com; 3 Division of Prevention, San Maurizio Hospital, 13060 Bolzano, Italy; roberto.grigo@tiscali.it; 4 Division of Pathology, European Institute of Oncology IRCCS, 20132 Milan, Italy; fausto.maffini@ieo.it; 5 Division of Otolaryngology Head & Neck Surgery, European Institute of Oncology IRCCS, 20132 Milan, Italy; marta.tagliabue@ieo.it; 6 Division of Pathology, San Maurizio Hospital, 39100 Bolzano, Italy; rosa.negro@sabes.it; 7 Department of Neurosciences, Reproductive and Odontostomatological Sciences, Oral Medicine Unit, Federico II University of Naples, 80138 Naples, Italy; stefania.leuci@unina.it (S.L.); mignogna@unina.it (M.D.M.); 8 C.I.N.B.O. (Consorzio Interuniversitario Nazionale per la Bio-Oncologia), 66100 Chieti, Italy

**Keywords:** tongue, tongue squamous cell carcinoma, tongue surgery, neoplastic infiltration

## Abstract

Tongue squamous cell carcinoma is the most common malignancy in the oral cavity. Despite advances in diagnosis and treatment, the prognosis of advanced states has not significantly improved. Depth of invasion, pattern of invasion such as tumor budding grade, lingual lymph node metastasis in early stages, collective cell migration and circulating tumor cells in peripheral blood are some examples of the mechanisms that are currently receiving increasing attention in the evaluation of the prognosis of tongue cancers. Anatomic-based surgery showed that it is possible to improve loco-regional control of tongue cancer. In patients with a “T-N tract involvement”, there is significantly more distant recurrence (40%) in patients undergoing a compartmental tongue surgery. In general, the neoplastic infiltration of the lingual muscles is traced back to the finding of neoplastic tissue along the course of a muscle; however, the muscle fibers, due to their spatial conformation and the organization of the extracellular matrix, could influence the movement of tumor cells through the muscle, leaving its three-dimensional structure unchanged. We need to exclude the possibility that tongue muscle fibers represent a mechanism for the diffusion of cancer cells without muscle invasion.

## 1. Introduction

Tongue squamous cell carcinoma (SCC) is the most common malignancy in the oral cavity; studies show that oral squamous cell carcinoma in young nonsmokers originates primarily in the tongue [1]. Despite advances in diagnosis and treatment, the prognosis of advanced states has not significantly improved. Depth of invasion (DOI) [2], pattern of invasion such as tumor budding grade (TBG) [3], lingual lymph node metastasis in early stages [4], collective cell migration [5] and circulating tumor cells (CTCs) in peripheral blood [6] are some examples of mechanisms that are currently receiving increasing attention in the evaluation prognosis of tongue cancers. However, due to its muscular structure, the pattern of cancer tongue muscle invasion plays an important role in the progression of the disease. For example, Chandler et al. showed that local recurrence was higher with muscle invasion than DOI, although the positive predictive value of muscle invasion in regards to nodal status was slightly less than DOI [7]. Additionally, routes of tumoral spread in the oral tongue through muscle fibers should be taken into account to plan a complete resection through a standardized and replicable surgical technique [8]. Therefore, it is interesting to compare the aspects of tumor diffusion in lingual musculature on the basis of anatomical and surgical findings with the most recent basic research data [9].

## 2. Bedside to the Bench in Tongue Muscle Invasion: Arising Aspects from Gross Anatomy of the Tongue

The tongue can be considered a median muscle organ made up of two equal parts, separated from each other by the median raphe [10]. Tongue muscles of most mammals are divided into two main groups: the extrinsic (genioglossus, styloglossus, hyoglossus, and palatoglossus) and the intrinsic (superior and inferior longitudinal, transverse and vertical) [11]. The three-dimensional architecture of longitudinal muscle fibers of the tongue is very complex. Saito and Itoh, studying a rabbit tongue, found that the longitudinal muscle of the tongue as a whole had a three-dimensional mesh-like structure while the transverse and vertical muscles of the tongue entered this mesh-like structure of muscle bundles of the longitudinal muscle as flat muscle bundles [12]. The transverse and vertical muscles showed no ramification in the center of the tongue, where there is no longitudinal muscle [12]. These results were confirmed by Yujiro Sakamoto in a recent paper on structural arrangement of the intrinsic muscles of the human tongue [13].

Between the muscle fibers there are connective structures that include: the medium septum, a thick sheet of fascia in the midline of the tongue that serves as origin for the transverse muscle; the paramedian septum, fascia that separate the genioglossus muscle from the inferior longitudinal muscle and the lateral septum, and connective tissue that envelopes the inferior longitudinal muscle [14].

Epithelial–mesenchymal transition (EMT) of cancer cells and lymphogenesis of the tumor microenvironment are important events in tumor metastasis in patients with oral tongue squamous cell carcinoma [15]. Several studies highlighted the role of microenvironment in the development, progression and invasion of tumors; a variety of cell populations, such as cancer-associated fibroblasts and various infiltrating immune cells, and different molecular pathways are currently being studied [16]. In addition to the composition of the microenvironment, recent studies revealed that extracellular matrix (ECM) alignment is the most critical parameter in influencing cell morphology, polarization, and migratory behavior [17]. Studies revealed that cells and engaged tissue can be regarded as multi-component viscoelastic units, subject to reciprocal mechanochemical interactions that induce, guide or limit cell migration in a context-dependent manner [18]. A comprehensive understanding of tumor invasion into muscle tissue is lacking [9].

## 3. Bedside to the Bench in Tongue Muscle Invasion: Arising Aspects from Surgery Specimens

**Figure 1:**
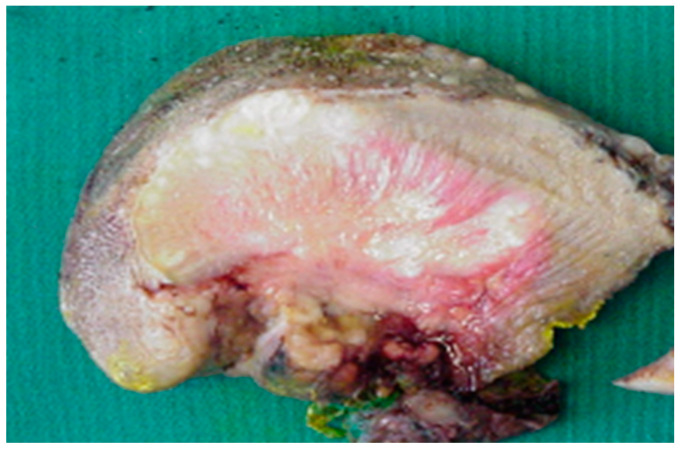
Surgical specimen of human tongue removed according to the principles of compartment surgery, demonstrating the spread of the disease in the portions closest to the epithelial surface and the propagation of the neoplastic thread along the course of the muscle fibers.

In our experience, the study of macroscopic anatomy of specimens from patients undergoing extensive excision of the tongue tissues and of the related anatomical components, allows the identification of sequential and specific stages of the diffusion of neoplastic infiltration (Figure 1). For simplicity, we will fundamentally analyze three phases: initial stages, late stages and the involvement of the N-T tract. Taking into account our observations of surgical specimens, in 2009 we developed the compartmental tongue surgery technique [19]. Since our original description, compartment surgery has been used by other authors [20,21].

(A) Initial stages

The anatomical analysis of the tongue surgical specimens of cancer patients showed that, in the initial stages, the tumor grows in the thin connective structures arranged between the muscle fibers, running parallel to the surface following the architectural texture of the intrinsic muscles. The spaces between the muscle fibers seem to guide the growth of the neoplasia. In our experience as surgeons, these spaces can be viewed as “low resistance anatomical” pathways and upon further analysis considered to constitute migration in confined spaces, where the structural characteristics of the extracellular matrix influence the diffusion of cancer cells. If this working hypothesis were correct, rather than “low resistance anatomical” pathways, these spaces could be construed as a preferential or obligatory path of diffusion of the disease in muscle tissue such as tongue tissue. Some recent research findings appear to corroborate this hypothesis. Cell adaptation to space and space availability together dictate migration efficacy [21]. Additionally, it is reported that cancer cells are less stiff than benign cells [20,22,23,24]. Wolf et al. reviewed how the ECM determines whether or not pericellular proteolysis is required for cell migration, ranging from non-proteolytic, non-destructive movement that solely depends on cell deformability and available tissue space to protease-driven invasion and secondary tissue destruction, in particular by matrix metalloproteinases (MMPs) and integrin- and actomyosin-mediated mechanic coupling [25,26]. Moreover, the study by Mathieu et al. revealed that cells migrate more efficiently upon increasing confinement and that for some cellular lines, the migratory potential of metastatic cells is superior compared to non-metastatic cells [27]. Interestingly, Sarkar et al. showed that the larger cell islands migrated more than the smaller islands on fibronectin. Smaller cell islands have a larger perimeter to area ratio and were expected to migrate more since migration occurs primarily from the periphery [28]. Cell migration along topographical features in the tissue microenvironment is clinically relevant in other tumors [29]. For example, local cell invasion from primary breast tumours is associated with bundled collagen fibers that align radially at the tumour–stroma interface [30].

(B) Late stages

**Figure 2:**
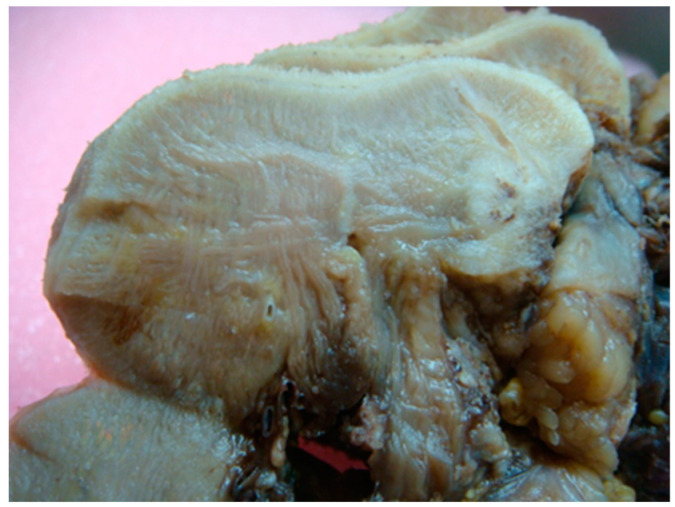
Intersection between intrinsic and extrinsic fibers tongue muscles.

Once cell tumors reach the transition zone with the extrinsic muscles (genioglossus, hyoglossus, styloglossus), the spread in the tongue always follows the longitudinal muscle fibers with cranio-caudal and anterior-posterior progression (Figure 2). In cases of advanced disease, the tumor progression is in accordance with the anatomy of the extrinsic muscles affected by neoplasia, extending up to reach the bone. A change in direction in neoplastic progression is observed in the crossing points between different fibers of extrinsic muscles, for example between hyoglossus and genioglossus, or, more frequently, between hyoglossus and mylohyoid, in the latter moving laterally towards the mandible.

**Figure 3:**
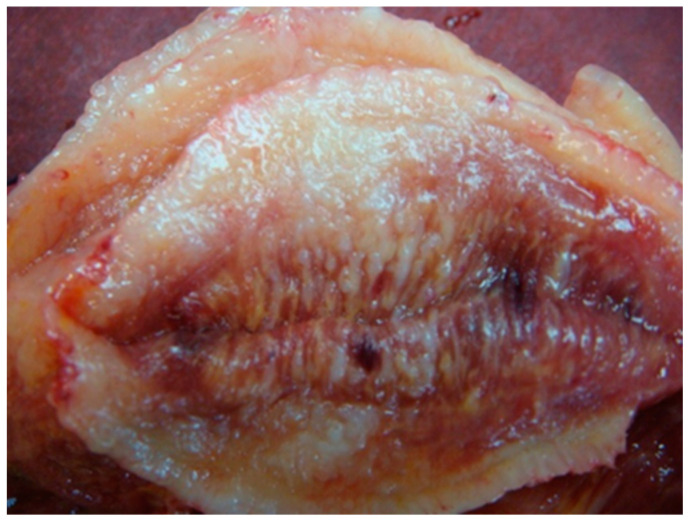
Longitudinal muscle progression of the tumor.

It is reported that in vivo, cells migrate through complex topographies that impose directional choices on cells. However, little is known about the inputs of a directional decision-making process in regions that present cells with different paths for cell migration. Some experimental evidence suggests that when cells reach an intersection, they ‘decide’ to follow the path of least hydraulic resistance, meaning that cells choose the shortest path or the path with wider microchannels [29]. However, we found longitudinal muscle progression of the tumour where the strong muscular activity pushes for a rapid tumor progression (Figure 3).

The tumour progression along the genioglossus muscles can come close to the septum but maintains a path parallel to it without going over it, always respecting the anatomical integrity, even if in contact. The overrun of the septum with the contra-lateral involvement only occurs along the intrinsic muscles above it. This is because the intrinsic muscles in this area constitute a unique structure that covers the dorsum of the tongue. Consequently, from a surgical point of view, since the lingual septum represents a cleavage and not a resection plane, the progression of neoplastic infiltration along the genioglossus muscle, even if close to the lingual septum, does not require radicalization of the contralateral genioglossus muscle. Even if the anatomical limit represented by the septum is exceeded, it is the anatomical characteristics of the lingual musculature that influence the progression of the contralateral compartment. The neoplasm can exceed the midline only above the lingual septum, spreading along the intrinsic transverse course muscle fibers. Thereafter, there may be an infiltration of the contralateral genioglossus musculature. This implies that, in the first case, a median-based wedge resection of the intrinsic musculature is necessary, while when the neoplasm begins to infiltrate the extrinsic musculature, a subtotal glossectomy is performed which also includes the removal of the contralateral genioglossus, leaving only a functional unit consisting of the tongue base and the hyoglossus muscle.

A distinctive feature of progression is the involvement of connective tissue located between the genioglossus and hyoglossus, where the vascular bundle of the hypoglossal nerve passes, and between the hyoglossus and mylohyoid, a connection area with latero-cervical lymph nodes containing the sublingual gland and lingual nerve.

**Figure 4:**
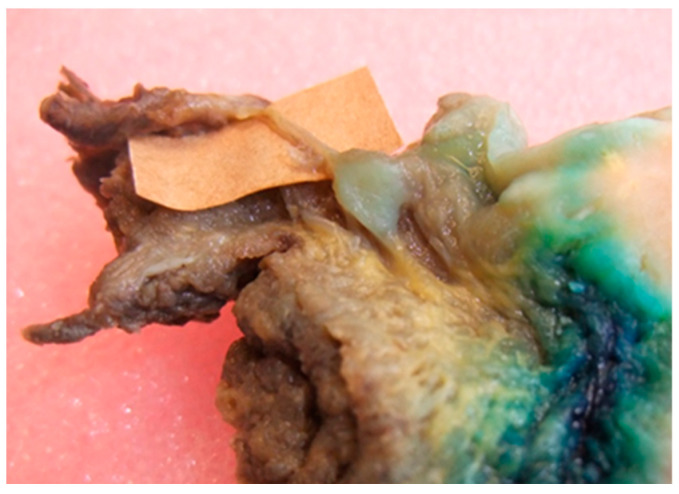
Metastasis along a nerve.

When the cancer reaches these areas, the nerves present with a diameter greater than 1 mm facilitate tumor progression along their axis with a perineural path [31] that accelerates tumor growth in the organ at a distance of a few centimeters (Figure 4). Perineural neoplastic deposits may arise individually along the nerve.

(C) The “T-N Tract”

We have defined the “T-N Tract” as the fibro-fatty tissues that connect the primary to the cervical lymphatic chain containing the neuro-vascular (lingual nerve and vein), glandular (sublingual gland) and lingual lymph nodes of the floor of the mouth localized on the internal surface of the mylohyoid muscle. The submandibular gland is considered part of the contents of Level IB of the neck and not part of the T-N tract.

Regional lymph node metastasis is a major prognostic factor for head and neck cancer because it indicates aggressive tumor biology, as well as representing a source of subsequent metastasis. A comprehensive discussion of the issues inherent in the involvement of cervical lymph nodes in tongue tumors goes beyond our objectives; in this context, we want to focus on the fact that, in addition to the structure of the lingual muscle bundles, it is possible to identify at least anatomically a district with a more lax collagen organization, which due to strategic relationships with numerous lymphatic and vascular structures and nerves plays an important role in the spread of tongue disease.

**Figure 5:**
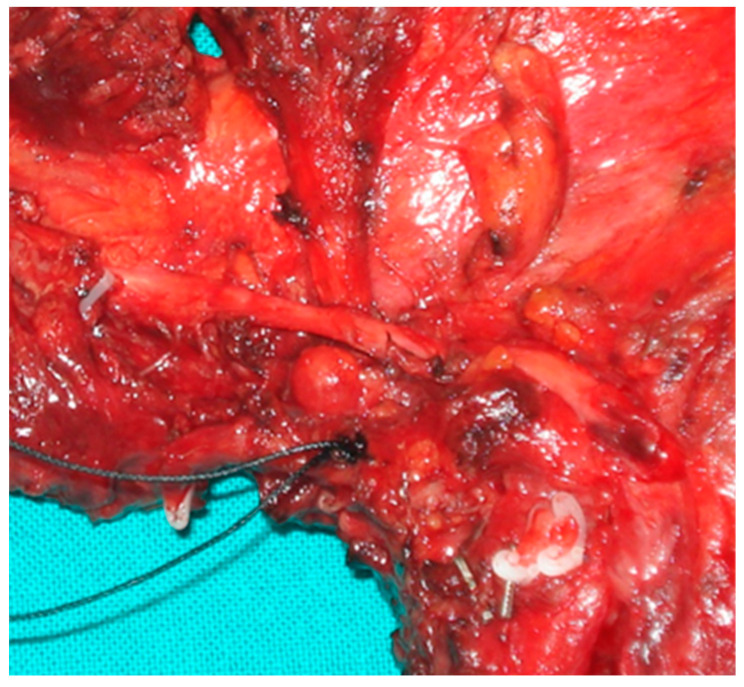
Lingual lymph nodes metastasis.

Regarding the patterns of diffusion, the analysis of the T-N tract showed the presence of metastatic lingual lymph nodes and lymphatic metastases “in transit” (Figure 5).

## 4. Discussion

We found that in the tongue, and contrary to some reports [32], the tumor behaved in an identical manner to tumors in musculoskeletal compartments of the extremities: tumor cell migration occurred longitudinally from the primary extending along and between the intrinsic and extrinsic muscle fibers, as noted by others [33,34], and progression was deflected by the anatomical boundaries of the compartment. In agreement with Shaheen [35], we also found that tumor progression occurred inferiorly (in depth) as well as posteriorly, following the sharp bend from the base of the tongue to the floor of the mouth. In this district, the course of nerves and vessels runs parallel to the muscle fibers and these structures are also likely pathways for tumor progression. Anatomical relationships therefore force tumor progression to take place longitudinally, de-emphasizing the circumferential approach to removing primary lesions. After 5 years, local disease control was achieved in 88.4% of compartimental tongue surgery (CTS) patients (16.8% improvement on standard surgery); locoregional disease control in 83.5% (24.4% improvement) and overall survival was 70.7% (27.3% improvement). The markedly improved outcomes in CTS patients, compared to those treated by standard surgery, indicate CTS as an important new approach in the surgical management of tongue cancer [36]. A recent study of Carta et al. pointed out that CTS performed on 80 patients with tongue carcinoma caused recurrence of the disease (local or lymphnode recurrences) in 18 patients (22.3%) [37].

A possible criticism of the compartmental approach in the treatment of lingual malignancies is whether improved survival is associated with poor functional outcomes. This aspect was investigated by Grammatica et al. in a recently published study. They showed that compartment surgery does not significantly affect speech. However, especially in the case of combined treatments, subclinical food aspiration and vallecular pouch were present in a significant percentage of patients. Finally, a correct reconstruction represents the key aspect for preserving the lingual strength [38].

In musculoskeletal or parenchymal locations, an “anatomical compartment” has been defined, where the fascia layers act as a barrier to primary tumor invasion, thus tumor spread is forced to follow the orientation of muscle fibers, parenchyma and soft tissues, including nerves and vessels. This is the reason why a single muscle, or a group of muscles, may be considered a “compartment”. Radical and complete removal of this anatomical unit is the aim of compartmental surgery [19].

Consequently, in the clinical setting, it is not possible to separate the effects of a single cellular or tissue activity from the other; for tongue cancer, it becomes necessary to consider other phenomena such as perineural invasion or involvement of regional lymph nodes, in particular lingual lymph nodes [4]. Today we can better plan how to perform tongue tumor removal as a result of improved knowledge of the manner of superficial and deeper local tumoral spread along the muscles, the nerves, and the vessels, and improved imaging techniques, which allow surgeons to evaluate the involvement of each tongue muscle.

To reinforce the idea that like smooth muscle invasion, neoplastic invasion of skeletal muscle is associated with worsened cancer prognosis, Beunk et al. [9] cited the work of Liao et al., which focused on how extrinsic muscle invasion should be considered for adequate patient staging [39]. It should be noted that it is the arrangement of the extrinsic muscles, with a sharp bend from the base of the tongue to the floor of the mouth, which facilitates fast progression of the tumor to the deeper tissue planes [36]. In this case, the biological behavior seems more to reflect the anatomic characteristics of the district than the mechanism of muscle invasion.

Undoubtedly, we need to improve our basic scientific knowledge and we need to improve our ability to translate as much of our basic scientific knowledge into significant disease altering therapeutic strategies in terms of local, loco-regional, functional and overall survival [40]. For example, in order to highlight the notion that it is time to reconsider our knowledge of human microscopic anatomy, Rosa et al. recently found a previously neglected cell population residing in the stromal space, namely telocytes, in interstitial meshworks of the tongue striated muscle and in the lamina propria. Telocyte locations within the stromal space of the normal human tongue could represent an important discovery for future investigations of TCs in different tongue pathologies [38]. On the other hand, genes related to muscle contraction have been reported to be altered in oral squamous cell carcinoma [41] and, in the study of Shaikhet al., gene ontology analysis found muscle contraction as the most enriched biological process in head and neck squamous cell carcinoma [42]. Additionally, the movement of collective cells requires the coordination of actomyosin organization between cells. Actomyosin contractility is high around the edge of the cell cluster and low between cells [43]. Additionally, Gopal et al. identified oncofetal fibronectin (FN) as a major and obligate component of the matrix assembled by stromal fibroblasts from head and neck squamous cell carcinomas (HNSCC) [44]. Moreover, Saénz-de-Santa-María et al. showed that the presence of “leader” mesenchymal cancer cells or “leader” fibroblasts was significantly associated with metastasis development, recurrent disease and low overall disease survival in head and neck squamous cell carcinomas [45]. On the other hand, whether cancer cells in lymph nodes can seed distant metastases has been a subject of considerable debate. Pereira et al. found that in mouse models, lymph node metastases can be a source of cancer cells for distant metastases [46]. The recent study by Hamada et al. proved the usefulness of a combined assessment of a histological picture, as an expression of the neoplastic disease at a given moment (surgical resection), and the expression of molecular markers as indicators of invasion mechanisms [47]. These authors found that in patients with low tumor budding grade, considered (histological) morphometric findings of invasiveness, the presence of podoplanin was a significant predictor of neck lymph node metastasis.

From this perspective, the study by Beunk et al. allows us to deepen our knowledge of tumor spread in tumor tissue and to fill a large gap in this field [9]. In fact, even if the path of cancer diffusion through the tongue muscle reflects the anatomical relationship among them, we need to find new answers to other problems, to explain, for example, a significantly more distant recurrence (40%) in patients undergoing a compartmental tongue surgery with “T-N tract involvement” [48]. Interestingly, as previously reported in the text, Chandler et al. showed that local recurrence was higher with muscle invasion than DOI, although the positive predictive value of muscle invasion regarding nodal status was slightly less than DOI [7].

It was reported that clusters of CTCs carrying an elevated risk of metastatic lesions at distant sites and their presence in the peripheral circulation of cancer patients are generally associated with a poor prognosis [49]. Additionally, Hidalgo-Carcedo et al. showed that collective movement requires the coordination of actomyosin organization between cells [43]. Could diffusion through tongue fibers muscles facilitate the diffusion of CTCs? Interestingly, clusters of CTCs include homotypic clusters made up of cancer cells only, as well as heterotypic clusters that incorporate stromal or immune cells along with cancer cells [50]. The study by Kulasinghe et al., in addition to showing that CTC clusters are found in locally advanced patients, speculated that leukocyte involvement in CTC heterotypic clusters may have a clinical relevance [51]. The fact that CTCs are found in the late stage and the possible role of leukocyte involvement in formation CTCs could make it possible to hypothesize a link. Interestingly, some studies showing an elevated pre-treatment neutrophil-lymphocyte ratio (defined as the absolute neutrophil count divided by the absolute lymphocyte count), were associated with a shorter locoregional recurrence but not with disease survival [52]. However, contrary to late dissemination, Werner-Klei et al. showed that melanoma cells leave primary tumors early and evolve at different parallel sites [53]. Similar results were reported by Rhim et al. in a mouse model of pancreatic cancer. Tagged cells invaded and unexpectedly entered the bloodstream early, before frank malignancy. This behavior was widely associated with epithelial-to-mesenchymal transition (EMT) [54]. Among typical features of epithelial–mesenchymal transition of tongue carcinoma, there is the expression of alpha smooth muscle actin. Could these observations raise the possibility that, through a muscular mechanism, the structural organization of tongue muscles could facilitate the early spread of cells outside of the tongue compartment?

## 5. Conclusions

Anatomic-based surgery showed that it is possible to improve loco-regional control of tongue cancer. However, in patients with a “T-N tract involvement”, there is a significantly more distant recurrence (40%) in patients undergoing a compartmental tongue surgery. In general, the neoplastic infiltration of the lingual muscles is traced back to the finding of neoplastic tissue along the course of a muscle; however, the muscle fibers, due to their spatial conformation and the organization of the extracellular matrix, could influence the movement of tumor cells through the muscle, leaving its three-dimensional structure unchanged. We need to exclude the possibility that tongue muscle fibers represent a way for diffusion of cancer cells without muscle invasion.
